# Identification of RNA-binding protein genes associated with renal rejection and graft survival

**DOI:** 10.1080/0886022X.2024.2360173

**Published:** 2024-06-14

**Authors:** Zhaozhong Zhong, Yongrong Ye, Liubing Xia, Ning Na

**Affiliations:** Department of Kidney Transplantation, The Third Affiliated Hospital of Sun Yat-sen University, Guangzhou, China

**Keywords:** RNA-binding proteins, renal transplantation, renal rejection, graft loss, prognostic model

## Abstract

Rejection is one of the major factors affecting the long-term prognosis of kidney transplantation, and timely recognition and aggressive treatment of rejection is essential to prevent disease progression. RBPs are proteins that bind to RNA to form ribonucleoprotein complexes, thereby affecting RNA stability, processing, splicing, localization, transport, and translation, which play a key role in post-transcriptional gene regulation. However, their role in renal transplant rejection and long-term graft survival is unclear. The aim of this study was to comprehensively analyze the expression of RPBs in renal rejection and use it to construct a robust prediction strategy for long-term graft survival. The microarray expression profiles used in this study were obtained from GEO database. In this study, a total of eight hub RBPs were identified, all of which were upregulated in renal rejection samples. Based on these RBPs, the renal rejection samples could be categorized into two different clusters (cluster A and cluster B). Inflammatory activation in cluster B and functional enrichment analysis showed a strong association with rejection-related pathways. The diagnostic prediction model had a high diagnostic accuracy for T cell mediated rejection (TCMR) in renal grafts (area under the curve = 0.86). The prognostic prediction model effectively predicts the prognosis and survival of renal grafts (*p* < .001) and applies to both rejection and non-rejection situations. Finally, we validated the expression of hub genes, and patient prognosis in clinical samples, respectively, and the results were consistent with the above analysis.

## Introduction

Currently, kidney transplantation is the main treatment for end-stage renal disease, providing patients with a better quality of life [1,2]. However, the long-term prognosis of kidney transplant recipients remains an obstacle, with graft rejection being one of the main causes [3,4]. Timely recognition and aggressive treatment of rejection are essential to prevent disease progression. Monitoring indicators other than renal puncture biopsy lack specificity, so the search for new noninvasive biomarkers has high application value [5].

In recent years, post-transcriptional regulation of genes has attracted much attention [6,7]. RNA-binding proteins (RBPs) bind to target RNA to form ribonucleoprotein complexes, which play a critical role in post-transcriptional gene regulation (PTGR) by affecting RNA stability, processing, splicing, localization, transport, and translation, thereby regulating gene expression [8–10]. More than 1500 RBPs have been found, and their expression is found in almost all cells [11]. Dysregulation of RBPs is strongly associated with many renal diseases, including renal clear cell carcinoma, renal ischemia/reperfusion injury, diabetic nephropathy, renal fibrosis, etc. [12–16]. However, RBPs have been little studied in renal transplantation, and it is not yet clear how RBPs related genes are involved in graft rejection and whether they have the potential to serve as prognostic markers for graft loss. The aim of this study was to comprehensively analyze the relationship between RBPs and renal rejection and to construct a prediction model for long-term renal transplantation outcomes based on RBPs. Initially, we identified differentially expressed RBPs (DE-RBPs) in kidney rejection patients using data from the GEO database and all RBP genes. The rejection related hub genes were then further identified by various machine learning methods. Based on hub DE-RBPs, kidney transplant patients were categorized into two clusters with different molecular characteristics. In addition, we constructed robust models capable of diagnosing T cell mediated rejection (TCMR) and predicting the long-term prognosis of kidney transplantation. Finally, we also validated them in clinical kidney transplant specimens. Notably, this is the first study to establish patient categorization and construct predictive features for long-term graft survival based on RBP genes expression in kidney transplantation.

## Materials and methods

### Data collection

The microarray and RNA-seq datasets used in this study were obtained from the GEO database (https://www.ncbi.nlm.nih.gov/geo/). A total of four datasets were included in this study, details of which were shown in Table S1. The GSE36059 dataset was used to analyze gene differential expression profiles in renal rejection and non-rejection groups and to construct diagnostic models. GSE25902 and GSE48581 were used to validate gene expression and validate diagnostic models. The GSE21374 dataset was used for subsequent predictive model construction. Gene symbols were obtained using the matching platform file. All datasets were normalized and log2 transformed using the ‘limma’ R package. The RBP genes used in this study were obtained from previous studies [11].

### Identification and validation of DE-RBPs

**Figure 1.**
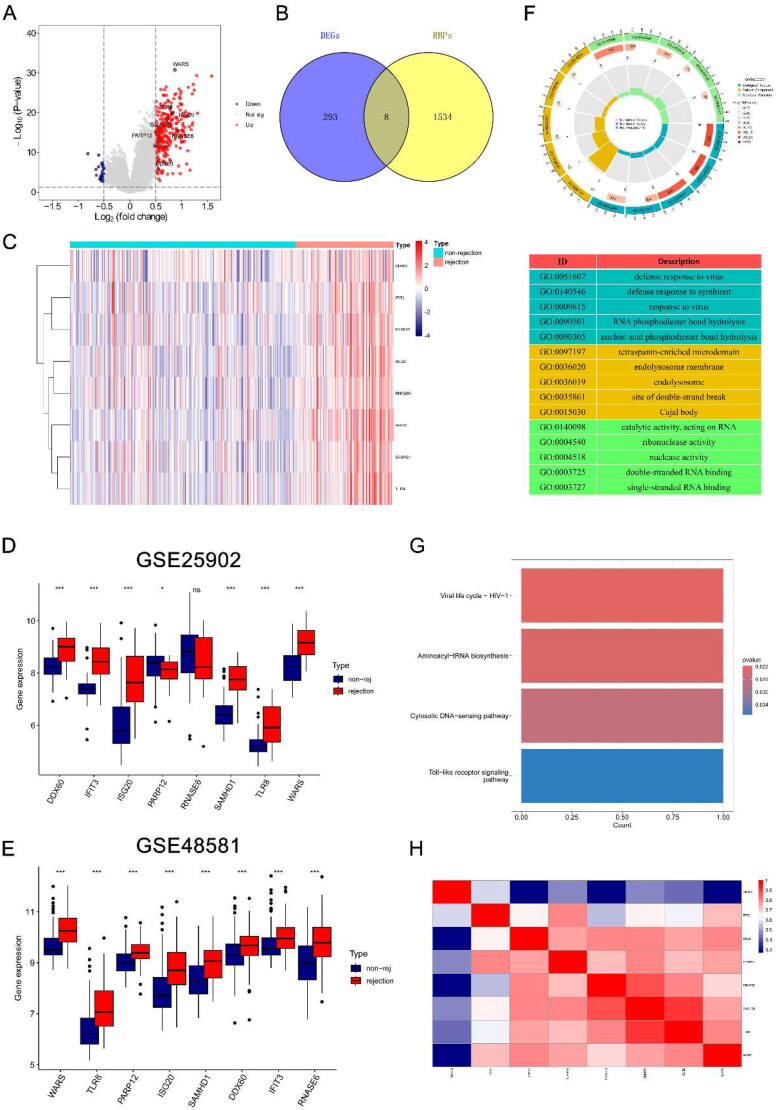
Identification of DE-RBPs and functional enrichment analysis. (A) Volcano plot showing differentially expressed RBPs, DE-RBPs are circled and labeled. (B) Intersection of DEGs and RBPs in renal rejection. (C) Heatmap showing the expression of DE-RBPs in the renal rejection and non-rejection groups. (D, E) Box plots showing expression of DE-RBPs in GSE25902 and GSE48581. (F) GO analysis reveals enrichment of DE-RBPs in biological processes, cellular components, and molecular functions. (G) KEGG pathway analysis of DE-RBPs. (H) Spearman’s correlation analysis showing the correlation of DE-RBPs. *p < .05; ***p < .001; ns: no significance.

The GSE36059 dataset containing 122 renal rejection samples and 281 non-rejection samples was used for the differential expression analysis between rejection and non-rejection. The samples were analyzed for differential expression using the ‘limma’ package to identify differentially expressed genes (DEGs). *p* Value <.05 and |logFC| >0.5 were set as thresholds. DEGs were then intersected with RBPs, resulting in the identification of DE-RBPs. DE-RBPs were obtained by taking the intersection of DEGs and RBPs, which has been shown in the Venn network (Figure 1(B)). Volcano plot and heatmap showed the expression of DE-RBPs. The GSE25902 and GSE48581 datasets were set as two independent cohorts to further validate the expression of DE-RBPs in rejection. Spearman’s correlation was employed to assess the correlation among DE-RBPs.

### Identification of rejection related hub genes by machine learning methods

To construct a predictive model for renal rejection, we used two machine learning methods to screen for transplant rejection related genes. The Random Forest (RF) algorithm, utilizing the ‘randomForest’ R package, and the support vector machine recursive feature elimination (SVM-RFE) algorithm, based on the ‘e1070’ R package, were used. The intersection of genes obtained from both algorithms identified eight hub RBPs associated with rejection, visualized in a Venn diagram. Subsequently, the DALEX package was used to test each model, obtaining cumulative residual distribution, and evaluating predictive performance using receiver operating characteristic (ROC) curves and area under the curve (AUC).

### Consensus clustering analysis

To identify the different RBP gene-associated renal transplantation subtypes, we performed a cluster analysis of all samples in the GSE21374 dataset using the R package ‘ConsensusClusterPlus’, where clustering was based on the expression of eight hub DE-RBPs. The ‘Partition Around Medoids’ algorithm, using Pearson’s distance to estimate sample similarity, was applied. The resampling method was applied to sample 80% of patients for 50 times. All samples were clustered into *k* (2–9) groups, determining the optimal number of clusters based on the cumulative distribution function (CDF) and Δ(*k*). Moreover, the principal component analysis (PCA) was performed to analyze expression differences between clusters.

### Functional enrichment analysis

The ‘clusterprofiler’ R package was used for Gene Ontology (GO) and Kyoto Encyclopedia of Genes and Genomes (KEGG) functional enrichment analysis, exploring functions and pathways of DEGs. Additionally, the ‘GSVA’ R package was used to analyze KEGG pathway activities for different clusters in GSE21374. The ‘clusterprofiler’ R package was also used for GO and KEGG functional enrichment analysis between different gene clusters. *p* Value <.05 was as considered significant.

### Construction and validation of TCMR diagnosis prediction model

Based on eight hub RBPs, the GSE36059 dataset was used to build a TCMR diagnostic model. The samples were randomly divided into a training set and an internal test set in a 1:1 ratio. The LASSO regression algorithm was used to build the final model. After deriving the coefficient values, the risk was calculated based on the RBPs expression and its corresponding regression coefficients. The sample of kidney transplant recipients was then grouped according to the median risk score. In addition, GSE25902 and GSE48581 were used as validation sets to test the robustness of the model. The heatmap shows the expression levels of the candidate genes in the model and the distribution of patients according to the risk score. ROC curves were used to assess the predictive power of the model.

### Construction of long-term survival predictive signature of allograft

Based on eight hub RBPs, the GSE21374 dataset was used to establish predictive signature for long-term allograft survival. Samples were randomly divided into training and testing sets at a ratio of 1:1. The LASSO regression algorithm was employed to build the final model. After the coefficient values are derived, the risk is calculated based on the RBP expressions and their corresponding regression coefficients. The sample of kidney transplant recipients was then grouped according to the median risk score. Kaplan–Meier (K–M) survival curves were used to make comparisons of survival between groups, and ROC curves were used to identify the predictive power of signature.

### Immune cell infiltration

Long-term survival predictive signature categorized the kidney transplant samples in GSE21374 into high and low risk groups. Using the ssGSEA, the infiltration of 23 immune cell types between high and low risk groups was compared. Additionally, CIBERSORT calculated immune cell infiltration levels, and the Spearman rank correlation coefficient evaluated the correlation between RBP genes and immune cells. *p* < .05 was considered statistically significant.

### Patients’ samples

Fourteen renal rejection and 17 non-rejection tissue samples were obtained from renal transplant recipients who underwent puncture at the Third Affiliated Hospital of Sun Yat-sen University, Guangzhou, China, and all included patients gave informed consent. Specimen collection and all experiments were approved by the Research Ethics Committee of the Third Affiliated Hospital of Sun Yat-sen University (II2024-052-01).

### Immunohistochemical staining

Paraffin-embedded renal biopsy tissue sections were baked at 65 °C for one hour, then deparaffinized three times in xylene and rehydrated in 100%, 95%, and 75% ethanol solution and distilled water. Peroxidase was blocked with 3% hydrogen peroxide solution for 10 min, and then the slides were boiled in an autoclave filled with citric hydrochloric acid (pH 6.0) for 25 min to repair the antigen. The sections were then blocked with 10% goat serum for 30 min. Then, the tissue sections were incubated with rabbit polyclonal anti-SAMHD1 (1:100 dilution, Genxspan, aa431–480) and WARS (1:100 dilution, Genxspan, aa35–84) primary antibodies overnight at 4 °C, followed by incubation with enzyme-labeled secondary antibodies for 45 min at 37 °C. DAB (3,3-diaminobenzidine) staining for 1.5 min was used for target protein detection. Image-Pro Plus software 6.0 was used to perform immunohistochemical analysis. To be specific, three randomly selected areas were observed in immunohistochemical staining sections using Image-Pro Plus software 6.0. Measure the total integrated optical density (IOD Sum) representing the intensity of immunohistochemical staining as well as the total area (Area Sum). Then calculate the mean density, mean density = IOD Sum/Area Sum. Finally, the average value was taken as the mean integrated optical density of the area.

### Statistical analysis

Statistical analyses were performed using GraphPad Prism software version 8 (La Jolla, CA) and R software 4.3.1 (R Foundation for Statistical Computing, Vienna, Austria). Differences between the two groups were compared using Student’s *t*-test, and *p* < .05 was considered statistically significant.

## Results

### Identification of DE-RBPs and functional enrichment analysis

**Figure 2.**
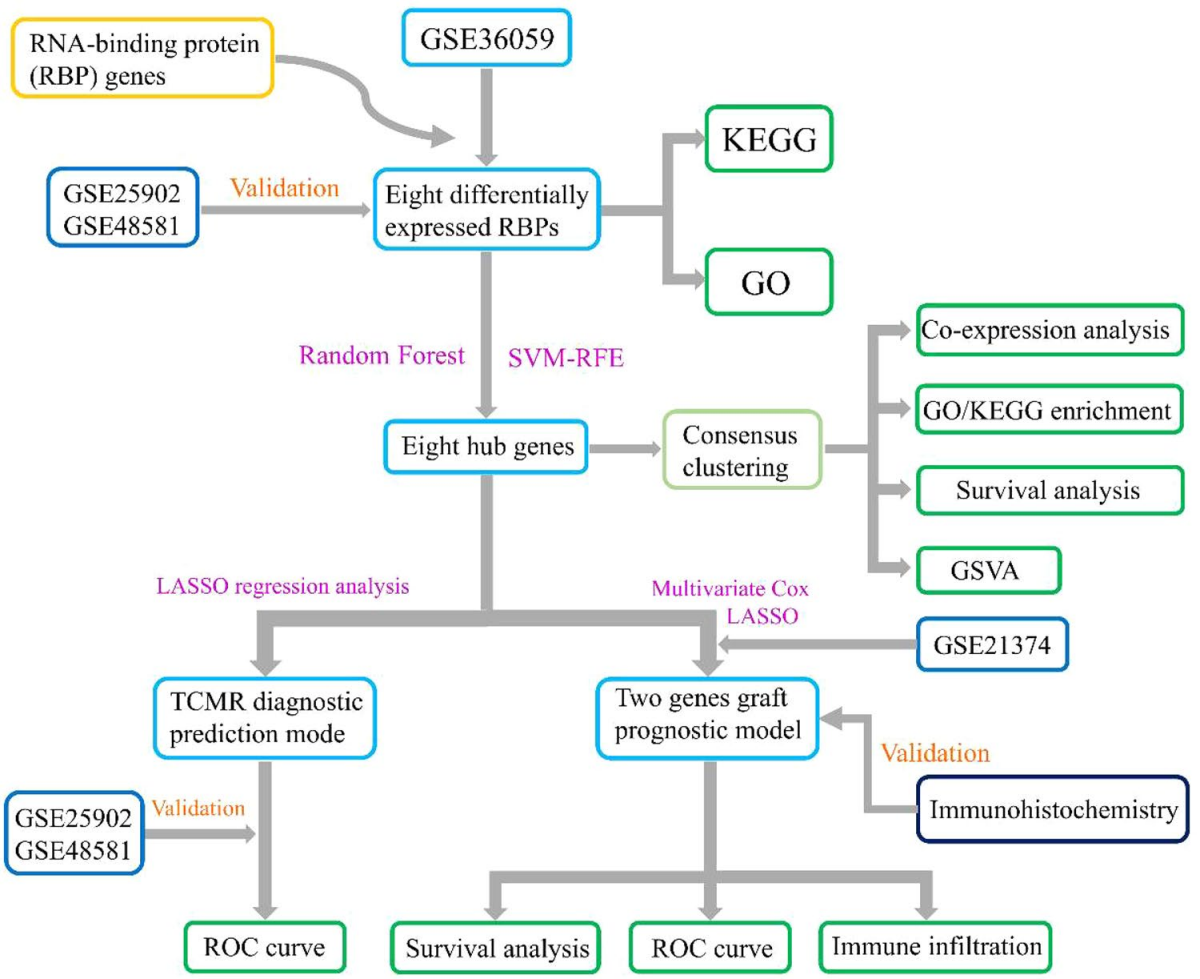
Flowchart of design of this study. RBPs: RNA-binding proteins; KEGG: Kyoto Encyclopedia of Genes and Genomes; GO: Gene Ontology; SVM-RFE: support vector machine recursive feature elimination; GSVA: gene set variation analysis; TCMR: T cell mediated rejection; LASSO: least absolute shrinkage and selection operator; ROC: receiver operating characteristic.

The experimental design and flowchart are depicted in Figure 2. Differential expression analysis using the GSE36059 dataset identified 301 DEGs (Figure 1(A)). Intersecting these with 1542 RBPs resulted in eight DE-RBPs (Figure 1(B), Table S2). Figure 1(C) illustrates their up-regulation in rejection. To further explore the expression of these eight DE-RBPs in rejected tissues, two independent cohorts were used to perform validation. As shown in Figure 1(D,E), these DE-RBPs were also markedly overexpressed in other cohorts of renal rejection tissues. GO analysis showed that DE-RBPs were related to defense response to virus, RNA phosphodiester bond hydrolysis, and nucleic acid phosphodiester bond hydrolysis (Figure 1(F)). The results of KEGG indicated that DE-RBPs were enriched in aminoacyl-tRNA biosynthesis, cytosolic DNA-sensing pathway, and Toll-like receptor (TLR) signaling pathway (Figure 1(G)). Spearman’s correlation analysis highlighted significant correlations among the eight DE-RBPs, particularly WARS, TLR8, SAMHD1, and RNASE6 (Figure 1(H)).

### Construction of rejection predictive model

**Figure 3.**
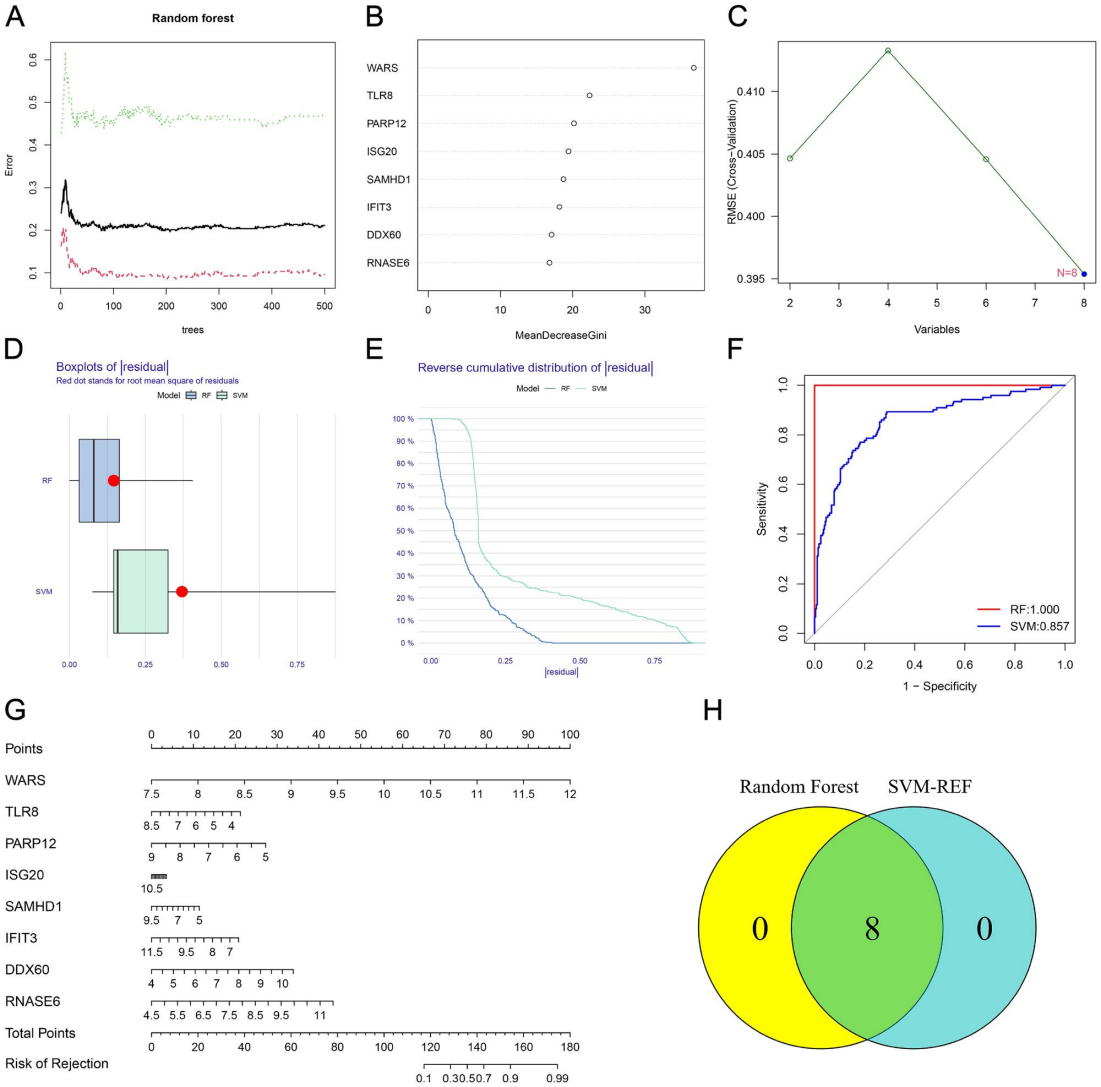
Establishment of renal rejection predictive model. (A) Random Forest Tree. Red represents renal rejection samples, green represents non-rejection samples, and black represents overall samples. (B) Gini importance measure. (C) The SVM-RFE algorithm selects feature RBPs at the optimal point. (D) Boxplot of residuals compares the two models. (E) Cumulative residual distribution plot compares the two models. (F) ROC curves compare the accuracy of the two models. (G) A nomogram model for predicting the risk of renal rejection based on DE-RPBs. (H) Random Forest and SVM-RFE screened hub RBPs take the intersection.

Renal rejection is an important risk factor for graft loss. Since these DE-RBPs are significantly differentially expressed in non-rejection and rejection groups, we tried to construct a rejection prediction model based on these genes. RBPs associated with renal rejection were identified by RF algorithm, and eight DE-RBPs were ranked according to the Gini importance measure (Figure 3(A,B)). The SVM-RFE algorithm was also employed to screen renal rejection related RBPs, and it had the smallest error when there were eight variable RBPs (Figure 3(C)). The ‘DALEX’ R package was used to analyze both models and the boxplots of residuals (Figure 3(D)). Based on the reverse cumulative residual distribution of residuals and AUC, RF (AUC = 1.000) was selected as the best model for diagnosing renal rejection (Figure 3(E,F)). Figure 3(G) shows the constructed nomogram model for predicting renal rejection. After intersecting the genes identified by RF and SVM-RFE algorithm, eight hub genes were finally obtained.

### Patient stratification based on hub DE-RBPs

**Figure 4.**
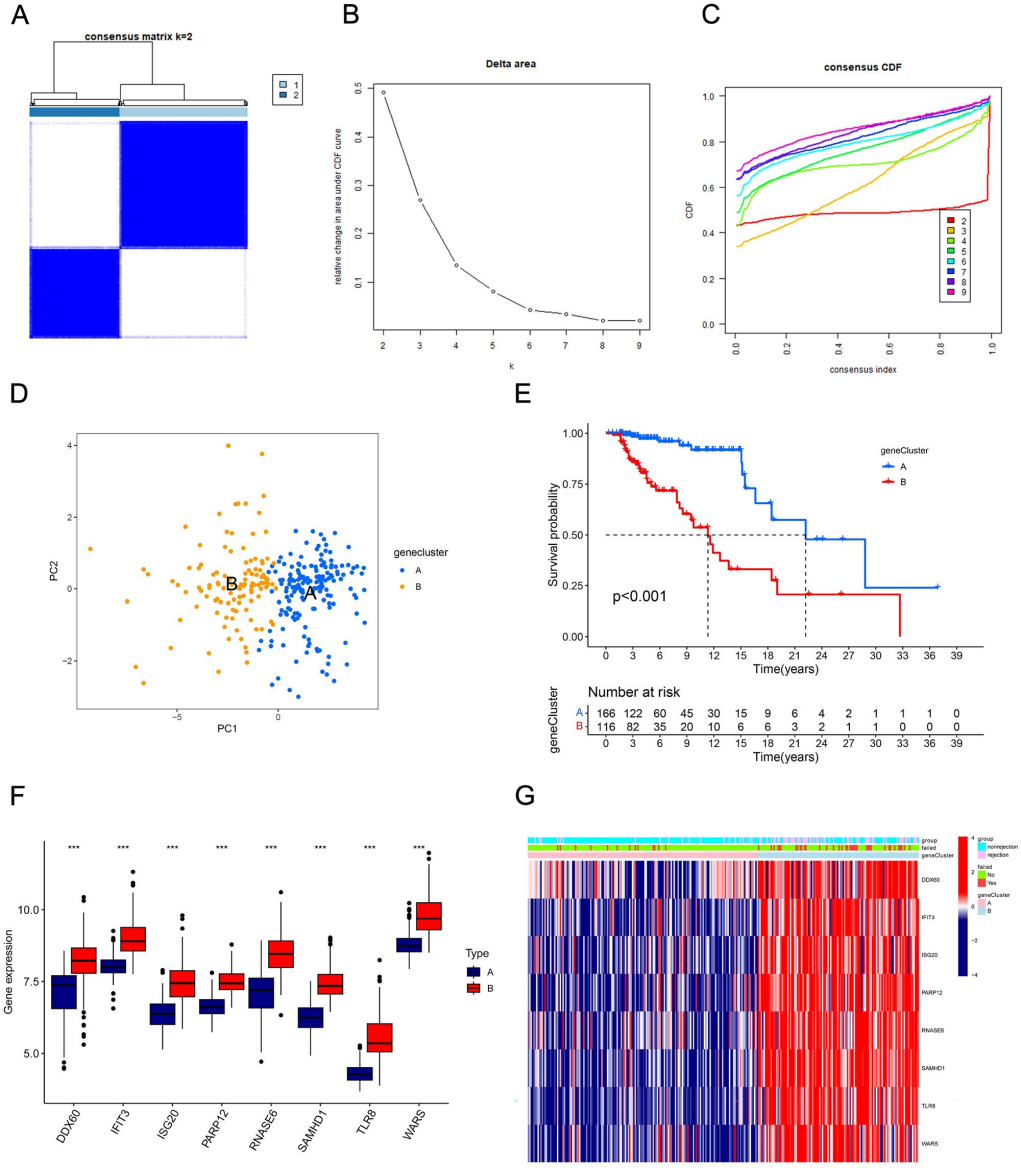
Transplant patients were categorized into two clusters according to hub DE-RBPs using consensus cluster analysis. (A) Consensus matrix of kidney transplant samples when *k* = 2. (B, C) Relative change in the area under CDF curve and consensus CDF when *k* = 2–9. (D) Principal component analysis (PCA) showed different expression patterns of the two gene clusters, A and B. (E) K–M survival analysis showed survival differences between the two gene clusters. (F) Box plot showing the expression of hub DE-RBPs between two gene clusters. (G) Heatmap showing the relationship between the expression of hub DE-RBPs and the clinical characteristics of patients in two clusters. ***p < .001.

Next, we further analyzed the stratification of all kidney transplant patients based on hub DE-RBPs using consensus clustering analysis. Results showed that *k* = 2 was the optimal value when the clustering was increased from *k* = 2 to *k* = 9, and the consensus CDF and delta area chart indicated that when *k* = 2, the clustering results of all samples had the best robustness (Figure 4(A–C)). Principal component analysis results showed significant differences in DE-RBPs between the two clusters (Figure 4(D)). In addition, cluster A and cluster B had significant differences in the distribution of risk scores (*p* < .001, Figure 4(E)). It can be seen that all the eight DE-RBPs had higher expression levels in cluster B (Figure 4(F)). Figure 4(G) reveals that there were more patients with kidney rejection and graft loss in cluster B.

### Functional enrichment analysis of gene clusters

GSVA was then used to functionally analyze two gene clusters to explore their biological characteristics. The heatmap results showed that the TLR signaling pathway, apoptosis, chemokine signaling pathway, B cell receptor signaling pathway, T cell receptor signaling pathway, and other signaling pathways related to inflammation and immune response were significantly activated in cluster B (Figure S1A). The KEGG results suggested that the genes between the two clusters differed in biological functions such as cytokine–cytokine receptor interactions, chemokine signaling pathways, phagocytosis, and intestinal immune network in IgA production (Figure S1B). GO results also revealed distinct differences between the two groups in activation of immune response, immune response activating signaling pathway, immune response regulating signaling pathway, leukocyte mediated immunity, and other biological processes related to immune rejection (Figure S1C). These results all suggest that the inflammatory and immune responses are more severe in cluster B than in cluster A, and thus its risk of renal transplant rejection and graft loss is higher.

### RBP gene based diagnostic prediction model for TCMR

Notably, the GSE25902 dataset only contains patients with TCMR and non-rejection. And in Figure 1(D), we can observe that most hub DE-RBPs are highly expressed in TCMR. We guessed that hub DE-RBPs might be highly expressed mainly in TCMR. So, we validated it in GSE36059 dataset. It was found that compared to other transplanted kidney samples, hub DE-RBPs indeed showed higher expression levels in TCMR (including mixed) (Figure S2A). Therefore, we tried to construct a diagnostic prediction model based on hub DE-RBPs that can distinguish TCMR. To this end, we randomly assigned the samples of GSE36059 to either the training set or the internal testing set (1:1). The LASSO regression analysis screened ISG20, RNASE6, SAMHD1, TLR8, and WARS as candidate genes for the diagnostic prediction model of TCMR, with coefficients of 0.14, 0.56, 0.31, 0.05, and 0.89, respectively (Figure S2B–D, Table S3). The risk score of each patient was calculated and the patients were categorized into high risk or low risk groups based on the median risk score. Subsequently, the predictive efficiency of this diagnostic model was evaluated using the ROC curves. The AUC of ROC curves for the training set and the internal testing set were 0.86 and 0.871, respectively (Figure S2F,H). To further verify its robustness, we used the GSE25902 and GSE48581 datasets as validation sets. The results showed that the AUC of validation sets were 0.792 and 0.711 (Figure S2J,L). The diagnostic prediction model has good diagnostic performance for TCMR. Figure S2E,G,I,K shows risk maps and expression heatmaps of identified DE-RBPs in each cohort.

### Construction and validation of a long-term graft survival predictive signature

In the biological function analysis described above, we found significant differences in signaling pathway activation for inflammation and immune response between the two different clusters. Meanwhile, cluster B obviously possessed worse long-term prognosis of grafts. Therefore, we tried to construct a signature based on hub DE-RBPs that could predict long-term survival of grafts.

**Figure 5.**
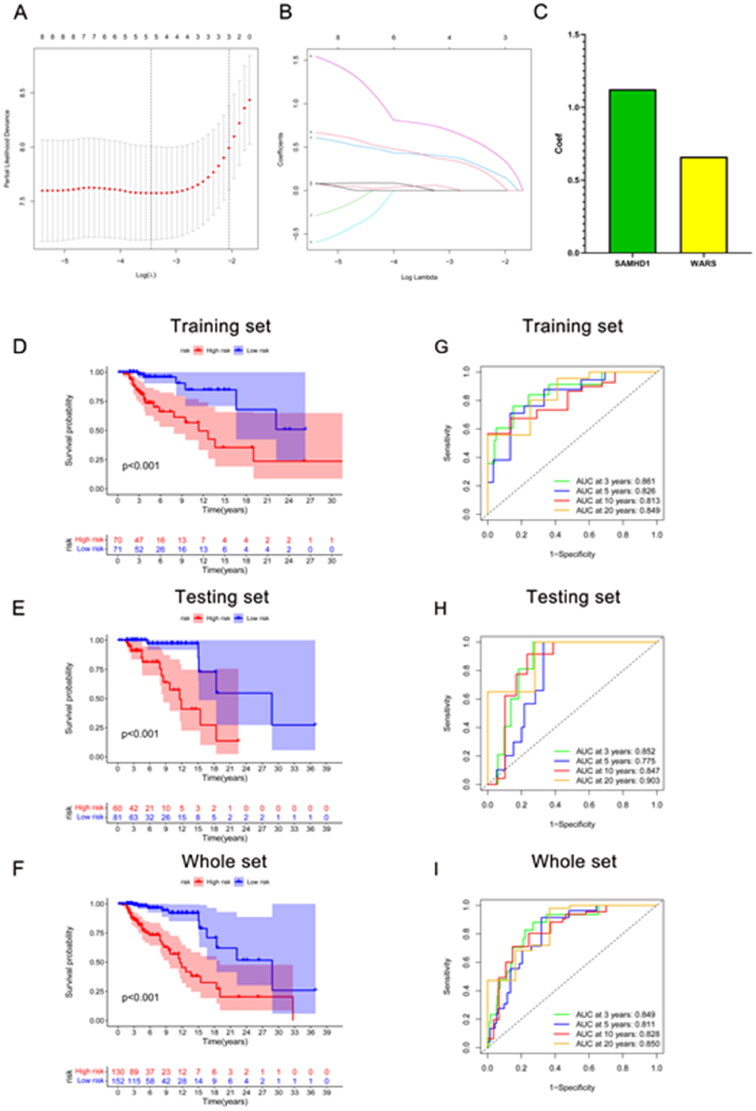
Construction of a predictive signature for long-term survival. (A, B) The 10-fold cross-validation LASSO obtained five candidate RBPs. (C) LASSO coefficients of candidate genes. (D–I) The signature’s K–M survival analysis and time-dependent ROC analysis on the training set, testing set, and whole set. LASSO: least absolute shrinkage and selection operator; K–M: Kaplan–Meier; ROC: receiver operating characteristic curve.

**Figure 6.**
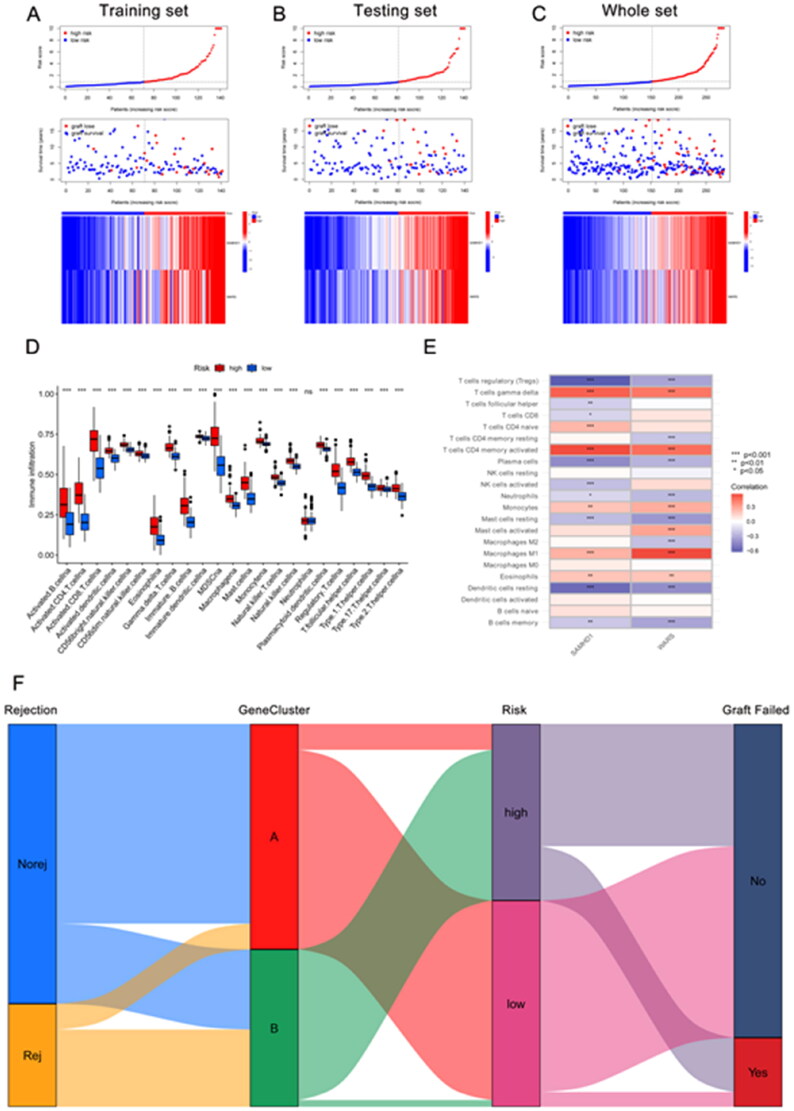
Immune infiltration analysis and risk map of long-term survival predictive signature. (A–C) Risk map of training set, testing set, and overall set. (D) Box plot showing differences in immune cell infiltration between low and high risk groups. (E) Correlation analysis between signature genes and infiltrating immune cells. (F) Sankey diagram showing the relationship between occurrence of rejection, gene clusters, recipient risk, and long-term graft failure. *p < .05; **p < .01; ***p < .001; ns: no significance.

**Figure 7.**
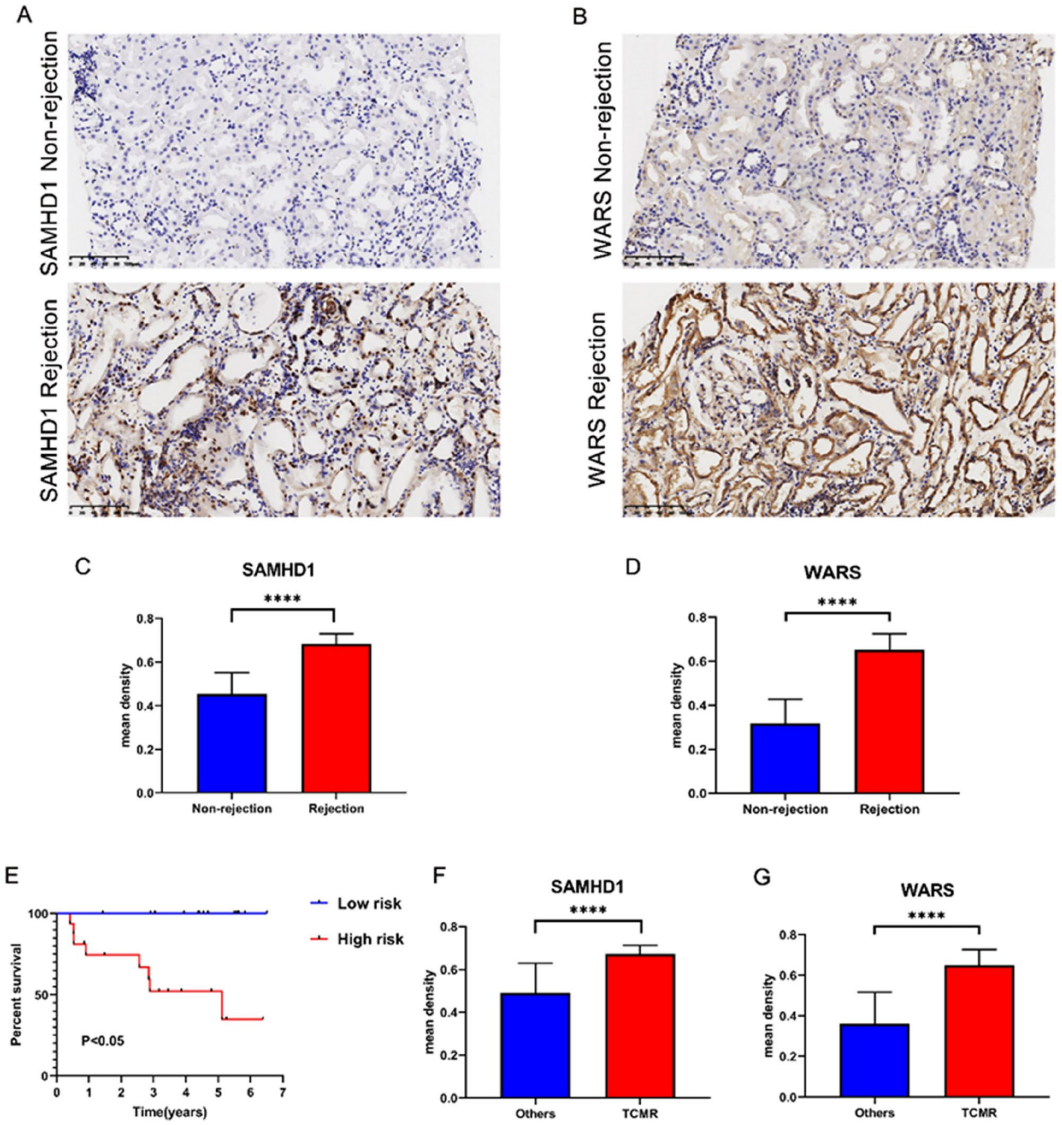
The expression of SAMHD1 and WARS in renal rejection. (A, B) Representative images of immunohistochemical staining of SAMHD1 and WARS in non-rejection and rejection samples. (C, D) Quantitative analysis of immunohistochemical staining of SAMHD1 and WARS in non-rejection and rejection samples. (E) K–M survival analysis in high and low risk groups. (F, G) Quantitative analysis of immunohistochemical staining of SAMHD1 and WARS in TCMR and other samples. ****p < .0001.

The kidney transplant patients in the GSE21374 dataset were randomly divided into a training set and a test set (1:1). Based on LASSO regression analysis, five potential genes (DDX60, IFIT3, PARP12, SAMHD1, and WARS) were screened for prediction modeling. Then, the relevant variables were further screened by multivariate Cox regression analysis. And the corresponding coefficients of each candidate gene were given to calculate the risk score of each sample. Finally, SAMHD1 and WARS were used to build the prognostic prediction model. The risk for each recipient was calculated as follows: Risk = 1.12 × Exp(SAMHD1) + 0.66 × Exp(WARS) (Figure 5(A–C),Table S4). K–M curves indicated that long-term graft survival was worse in the high risk group than in the low risk group (Figure 5(D–F)). ROC curves revealed that in all cohorts, the AUC was greater than 0.7 at years 3, 5, 10, and 20, suggesting that the model has good indicator for long-term graft survival (Figure 5(G–I)). Figure 6(A–C) shows that the expression of SAMHD1 and WARS was significantly higher in the high risk group, and also the recipients in the high risk group were more prone to graft loss. To further validate the expression of hub DE-RBPs, we collected some clinical kidney puncture samples for experimental verification. The results of immunohistochemistry showed that the expression of SAMHD1 and WARS was significantly higher in rejection samples than in non-rejection samples (Figure 7(A–D)). Patients were categorized into high or low risk groups based on the expression of SAMHD1 and WARS, and it was found that the low risk group had a better prognosis (Figure 7(E)). Table S5 shows some of the clinical characteristics of the two groups of patients. In addition, the expression of SAMHD1 and WARS in TCMR samples was significantly higher than that in other samples (Figure 7(F,G)). These results are all consistent with our analysis above. Sankey plots visualize the relationship between the occurrence of rejection, gene clusters, recipient risk, and long-term graft failure (Figure 6(F)).

### Immune infiltration analysis based on long-term graft survival predictive model

The immune response is a major cause of graft rejection and loss, and understanding cellular infiltration in the immune microenvironment of transplantation can help us to further understand the entire biological process of disease development. In this regard, we used ssGSEA to evaluate and analyze relevant samples. The results of immune infiltration analysis suggested that many immune related cells, which included B cells, CD4+ T cells, CD8+ T cells, dendritic cells, NK cells, macrophages, and monocytes were more enriched in the high risk group (Figure 6(D)). Further analysis of the relationship between hub DE-RBPs and immune-associated cells revealed that SAMHD1 and WARS were positively correlated with activated CD4+ T cells, gamma delta T cells, M1 macrophages, and negatively correlated with regulatory T cells, resting mast cells, resting dendritic cells, and plasma cells (Figure 6(E)). The above results suggest that the immune response and inflammation were more severe in the high risk group, resulting in poorer long-term survival of the grafts.

## Discussion

In recent years, with the development of immunosuppressive therapies, the incidence of rejection after renal transplantation has decreased significantly [17]. However, graft rejection remains the major cause of late graft loss, and timely detection and diagnosis of rejection is important and necessary for long-term graft survival [3,18]. The identification of new biomarkers may provide new insights into the diagnosis of rejection and long-term graft survival. RBPs play an important role in post-transcriptional regulation, and their dysregulated expression has been found to be associated with an increasing number of renal diseases. However, the roles and mechanisms of RBPs in kidney transplant rejection and long-term graft survival are unknown. Therefore, this study used a number of datasets downloaded from the GEO database to explore the relationship between RBPs and kidney transplant rejection and graft loss. First, we used the GSE36059 dataset to discover DEGs and extracted intersections with RBPs, resulting in eight DE-RBPs. Then, the expression of these genes was validated by two additional datasets. GO and KEGG analyses revealed the relevant signaling pathways mainly involved in these genes. Next, RF and SVM-RFE algorithms were used to select signature genes and construct rejection-related prognostic models. Besides, based on the screened hub DE-RBPs, a TCMR diagnostic model was constructed using LASSO regression analysis, and the model was validated in two additional datasets. Finally, a risk model for graft loss based on SAMHD1 and WARS was constructed in the GSE21374 dataset using LASSO and multivariate Cox regression analyses and validated in clinical samples. In conclusion, our results suggest that risk modeling accurately predicts the prognosis of renal transplant recipients and may help to improve the diagnosis and clinical management of patients.

Post-transcriptional gene regulation is essential for maintaining normal physiological cellular function, and mechanistically, each of these events is regulated by the formation of distinct ribonucleoprotein complexes centered on RBPs [11]. The occurrence and progression of an increasing number of diseases have been shown to be closely related to dysregulated expression of RBP. Some studies suggest that the disease phenotype of RBP may be associated with tissue-specific expression [19,20]. And in this study, by integrating the DEGs and RBPs, we obtained eight DE-RBPs that were highly expressed in renal transplant rejection tissues. Functional enrichment analysis showed that they were significantly enriched in defense response to virus, RNA phosphodiester bond hydrolysis, aminoacyl-tRNA biosynthesis, cytosolic DNA-sensing pathway, and TLR signaling pathway. BK virus infection is recognized as one of the causes of interstitial nephritis and allograft failure in kidney transplant recipients [21,22]. The degree of damage to renal tissue, inflammation of the organism in response to the virus, and subsequent fibrosis following viral infection influence the outcome of the transplanted kidney. Previous studies have shown that RBPs regulate the development of a wide range of diseases mainly through PTGR [23–25], and thus would involve RNA phosphodiester bond hydrolysis and biosynthesis. Toll-like receptors are one of the innate immune receptors capable of responding rapidly to the presence of non-autologous molecules, tissue damage, or tissue stress [26]. A large number of experimental and clinical studies have demonstrated that the TLR signaling pathway plays an important role in the development of inflammation and rejection of allografts [27–30]. Anti-inflammatory strategies targeting TLRs may improve the long-term prognosis of solid organ recipients [31]. Subsequently, we further identified hub genes using a machine learning algorithm and categorized patients into cluster A and cluster B based on these hub genes using consensus clustering analysis. All of these eight hub RBPs were upregulated in cluster B. And cluster B recipients with high expression of DE-RBPs obviously had worse prognosis. The heatmap in Figure 4(G) also shows that cluster B holds more kidney transplant rejection patients and renal failure patients. The results of GSVA analysis show that graft versus host and immune response related signaling pathways are significantly enriched in cluster B. GO and KEGG analyses show similar findings. These results suggest that RBPs can influence renal rejection by modulating multiple biological processes, thereby affecting long-term graft survival.

Previous studies have shown that the alloimmune processes are one of the main causes of graft loss, with TCMR and antibody mediated rejection (ABMR) being the most typical subtypes [32,33]. Among them, TCMR is the main early rejection phenotype and the endpoint of clinical trials [34]. Clinical diagnostic criteria for TCMR based on Banff classification are largely opinion-based and inconsecutive with arbitrary cutoffs, which have limitations [35]. Moreover, the histologic lesions of TCMR are not specific. For example, interstitial inflammation and tubulitis also occur in acute kidney injury and may lead to false positives, whereas scar tissue is difficult to assess and leads to false negatives [36]. In contrast, the advantages of molecular assessment over histologic methods include objectivity, reproducibility, and quantification, which can help improve pathologic diagnosis [35]. In this study, our model showed good diagnostic performance for TCMR. The AUCs of the internal testing set and the two external validation sets were 0.871, 0.792, and 0.711, which were basically similar to the diagnostic performance of m6A related immune gene based diagnostic prediction model for TCMR, whose AUCs of the internal testing set and external validation set were 0.891 and 0.854, respectively [37]. Our results suggest the potential application value of constructing a TCMR diagnostic model based on RBP genes to assist in clinical pathology diagnosis.

In addition, by LASSO analysis and multiple Cox regression analysis, we identified two hub RBPs (SAMHD1, WARS), and established a risk model for predicting graft survival. ROC curve analysis showed that our model was significant and sensitive, and was valuable in guiding the treatment and prognosis of renal transplant recipients. Among them, the AUC of risk score for 3 years of survival in the validation set is 0.852, which is better than NiNRG predictive model (AUC 0.735), which is a long-term graft survival prediction model constructed on five necroinflammation-associated necroptosis-related genes (NiNRG) [38]. In addition, our model also had better indications in long-term survival prediction at 10 years and 20 years. SAMHD1 was originally identified as being associated with autoimmune diseases [39]. Later, SAMHD1 was more frequently found to be associated with early immune responses to viruses [40–43]. Subsequently, more studies have identified the involvement of SAMHD1 in innate immunity, inflammatory response, and cancer development [44,45]; however, the role of SAMHD1 in renal rejection and long-term survival of transplanted kidneys has not yet been elaborated. SAMHD1 is predominantly found in the nucleus and thus was once considered a nuclear protein [46,47]. However, SAMHD1 is also detected in the cytoplasm. Nucleoplasmic shuttling of SAMHD1 may be important for SAMHD1 to fulfill different functions [48,49]. Our experimental results observed that SAMHD1 has high expression in the nucleus and some expression in the cytoplasm (Figure 6). This differential cellular localization may suggest a potential role in the rejection response and needs to be further explored. WARS, also known as WARS1, is a protein produced by IFNγ induction, and some of its fragments are vasopressor and therefore thought to play a role in vascular homeostasis [50,51]. Increased mRNA levels of WARS are reported to be associated with renal rejection [52]. Using a proteomic approach, one study found that high expression of the interferon associated protein WARS1 could be used as a specific indicator of endothelial stress along with TYMP and GBP1 to aid in the diagnosis of ABMR [53,54]. Our study also demonstrates its important potential diagnostic value in kidney transplant rejection and long-term prognosis.

However, this study still has some limitations. First, some vital clinical features such as ischemic time, recipient age, DGF, and viral infection status were not included. Incorporating these factors might improve the validity and reliability of the model. Second, the stability of this prediction model also needs more data sets to be validated. Meanwhile, although we validated the expression of RBPs in renal rejection in clinical samples, increasing the number of validation samples and further validating the relationship between RBPs and long-term prognosis of renal transplantation are necessary. Finally, the results of bioinformatics analysis presented in this study should be further explored and validated by *in vivo* and *in vitro* experiments.

In conclusion, based on GEO data, we performed a comprehensive bioinformatics analysis to investigate the prognostic value of aberrantly expressed RBPs in renal rejection. Ultimately, we developed a prognostic risk model containing two RBPs that can independently and accurately predict the prognosis of kidney transplant recipients. This study may help provide new insights and ideas for the diagnosis of rejection and long-term survival treatment strategies for kidney transplant recipients.

## Supplementary Material

Supplemental Material
