# Supplementary material for: Identification of RNA-binding protein genes associated with renal rejection and graft survival
Source: Ren Fail. 2024 Jun 14;46(2):2360173. doi: 10.1080/0886022X.2024.2360173 (PMC11182075; doi:10.1080/0886022X.2024.2360173)
Supplement: Supplemental Material [file IRNF_A_2360173_SM5687.docx]

**Table S1**: Description of datasets in this study.

| **GEO accession** | GSE36059 | GSE25902 | GSE48581 | GSE21374 |
| --- | --- | --- | --- | --- |
| **Platform** | GLP570 | GLP570 | GLP570 | GLP570 |
| **Sample tissue** | Kidney | Kidney | Kidney | Kidney |
| **Species** | Homo sapiens | Homo sapiens | Homo sapiens | Homo sapiens |
| **Sample size** | 403 | 120 | 306 | 282 |
| **Rejection** | 122 | 24 | 107 | 76 |
| TCMR | 35 | 24 | 29 | Unknown |
| ABMR | 65 | 0 | 32 | Unknown |
| Mixed ABMR and TCMR | 22 | 0 | 4 | Unknown |
| Borderline rejection | 0 | 0 | 42 | Unknown |
| **Non-rejection** | 281 | 96 | 199 | 206 |
| **Mean time from transplant to biopsy (month)** | 48 | 13 | 57 | 58 |
| **Indication for biopsy** |  |  |  |  |
| Primary nonfunction | 10 (2%) | unknown | 9(3%) | 0 |
| Deterioration of graft function | 246(61%) | unknown | 170 (57%) | 65(62%) |
| Stable impaired graft function | 71 (18%) | unknown | 17 (6%) | 7 (7%) |
| Investigate proteinuria | 38 (9%) | unknown | 71 (24%) | 15 (14%) |
| Follow-up from previous biopsy | 14 (3%) | unknown | unknown | 6 (6%) |
| Others | 9 (2%) | unknown | 17 (6%) | 6 (6%) |
| Indication unknown | 15 (4%) | 120(100%) | 16 (5%) | 6 (6%) |
| **Number of patients** | 315 | 120 | 264 | 105* |
| **Mean recipient age** | 43 (5–81) | 11(5.4-16.6) | 48(10-86) | unknown |
| **Recipient gender (% male)** | 196 (62%) | 69(57.5%) | 177 (67%) | 65(62%) |
| **Primary disease** |  |  |  |  |
| Diabetic nephropathy | 64 (20%) | unknown | 40 (15%) | 13(12%) |
| Hypertension/large-vessel disease | 29 (9%) | unknown | 9(3%) | 6 (6%) |
| Glomerulonephritis/vasculitis | 119 (38%) | 31(26%) | 65(25%) | 51 (49%) |
| Interstitial nephritis/pyelonephritis | 20 (6%) | 37(31%) | unknown | 8 (8%) |
| Polycystic kidney disease | 46 (15%) | 8(7%) | 30(11%) | 14 (13%) |
| Others or unknown | 37(12%) | 44(37%) | 120(46%) | 13(12%) |
| **Mean donor age** | 40 (2 –70) | 32(21-43) | 41(1-75) | unknown |
| **Donor gender (% male)** | 121 (46%) | 65(54%) | 100(38%) | 41 (39%) |
| **Donor type (% deceased donor transplants)** | 152 (49%) | 45(38%) | 163(61%) | 57 (54%) |
| **Failed grafts** | 80 | unknown | 33 | 30 |

**Table S2**: Differentially expressed RNA-binding proteins (DE-RBPs) with absolute log2 fold change (FC) > 0.5 and FDR < 0.05.

| **DE-RBPs** | **Log_2_ FC** | **P Value** | **FDR** |
| --- | --- | --- | --- |
| DDX60 | 0.55 | 1.82E-06 | 2.62E-05 |
| IFIT3 | 0.56 | 6.30E-14 | 4.53E-12 |
| ISG20 | 0.83 | 1.25E-20 | 4.53E-18 |
| PARP12 | 0.51 | 6.39E-16 | 7.06E-14 |
| RNASE6 | 0.91 | 2.72E-16 | 3.35E-14 |
| SAMHD1 | 0.73 | 3.58E-19 | 8.34E-17 |
| TLR8 | 0.82 | 1.21E-20 | 4.53E-18 |
| WARS | 0.87 | 1.69E-31 | 1.83E-27 |

**Table S3**: The corresponding coefficients of DE-RBPs in the diagnostic prediction model.

| **DE-RBPs** | **Coef** |
| --- | --- |
| ISG20 | 0.14 |
| RNASE6 | 0.56 |
| SAMHD1 | 0.31 |
| TLR8 | 0.05 |
| WARS | 0.89 |

**Table S4**: The corresponding coefficients of hub DE-RBPs in the prognostic prediction model.

| **DE-RBPs** | **Coef** |
| --- | --- |
| SAMHD1 | 1.12 |
| WARS | 0.66 |

**Table S5**: Clinical characteristics were comparable between low-risk and high-risk group.

| **Group** | **Low-risk** | **High-risk** |
| --- | --- | --- |
| **Gender** |  |  |
| Male | 11 | 7 |
| Female | 4 | 9 |
| **Mean age(SD), year** | 41.53(9.31) | 40.63(9.16) |
| **ESRD leading to transplant** |  |  |
| Glomerular disease | 4 | 2 |
| Polycystic disease | 2 | 0 |
| IgA nephropathy | 0 | 3 |
| Diabetes mellitus | 0 | 0 |
| Other | 0 | 2 |
| Unknown | 9 | 9 |
| **Diagnosis** |  |  |
| ABMR | 0 | 2 |
| TCMR | 2 | 13 |
| Non-rejection | 13 | 1 |
| **eGFR one week after transplantation(SD),**  **ml/min per 1.73 m^2^** | 25.73(22.71) | 42.63(21.24) |
| **eGFR one month after transplantation(SD),**  **ml/min per 1.73 m^2^** | 39.55(24.99) | 45.01(23.39) |


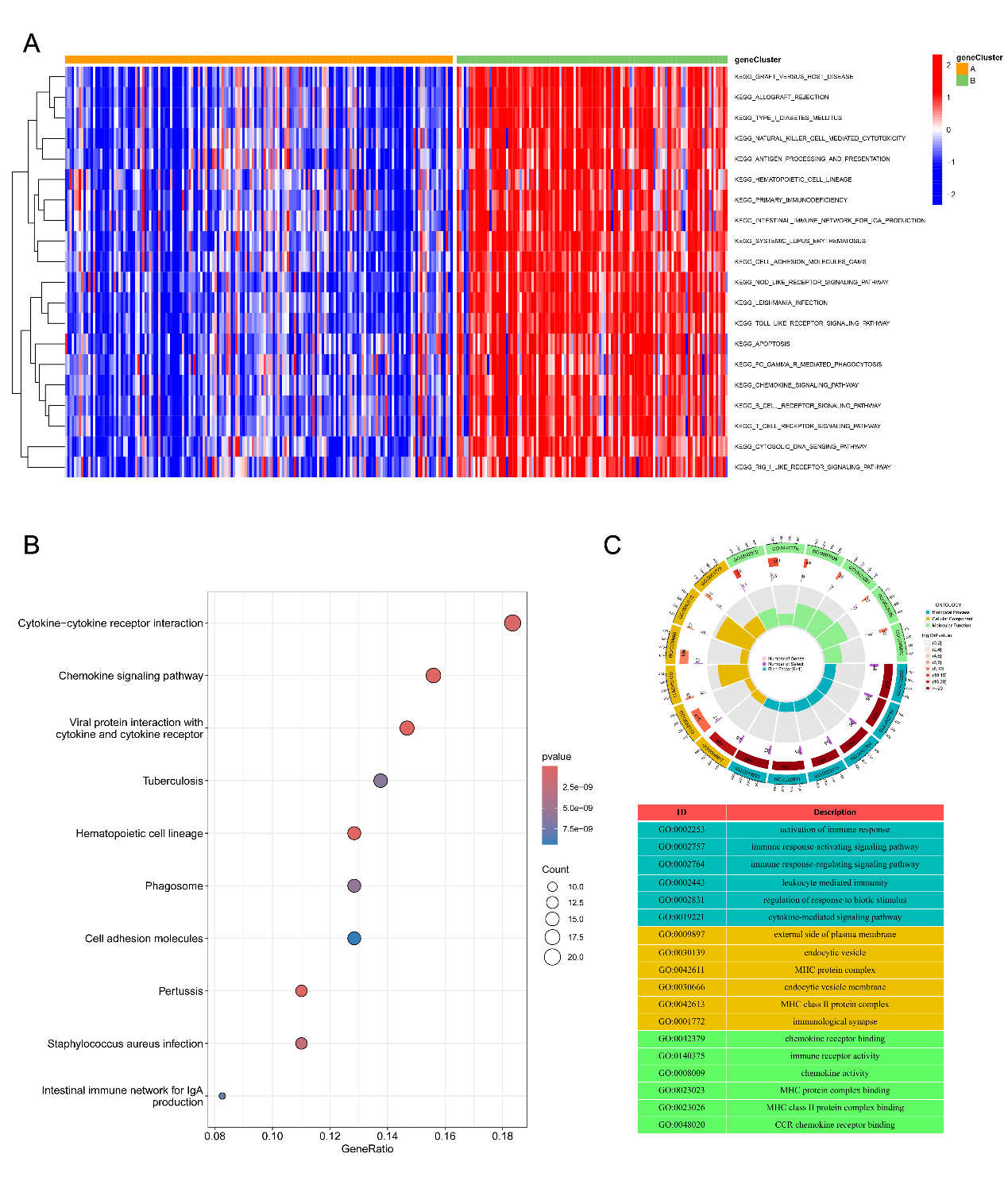


**Figure S1.** Functional enrichment analysis of kidney transplant clusters. (A) Heatmap showing the different characteristics between two clusters. (B, C) Differential genes between the two clusters were analyzed for GO and KEGG enrichment. GSVA, gene set variation analysis. P adjust <0.05 was considered significant.


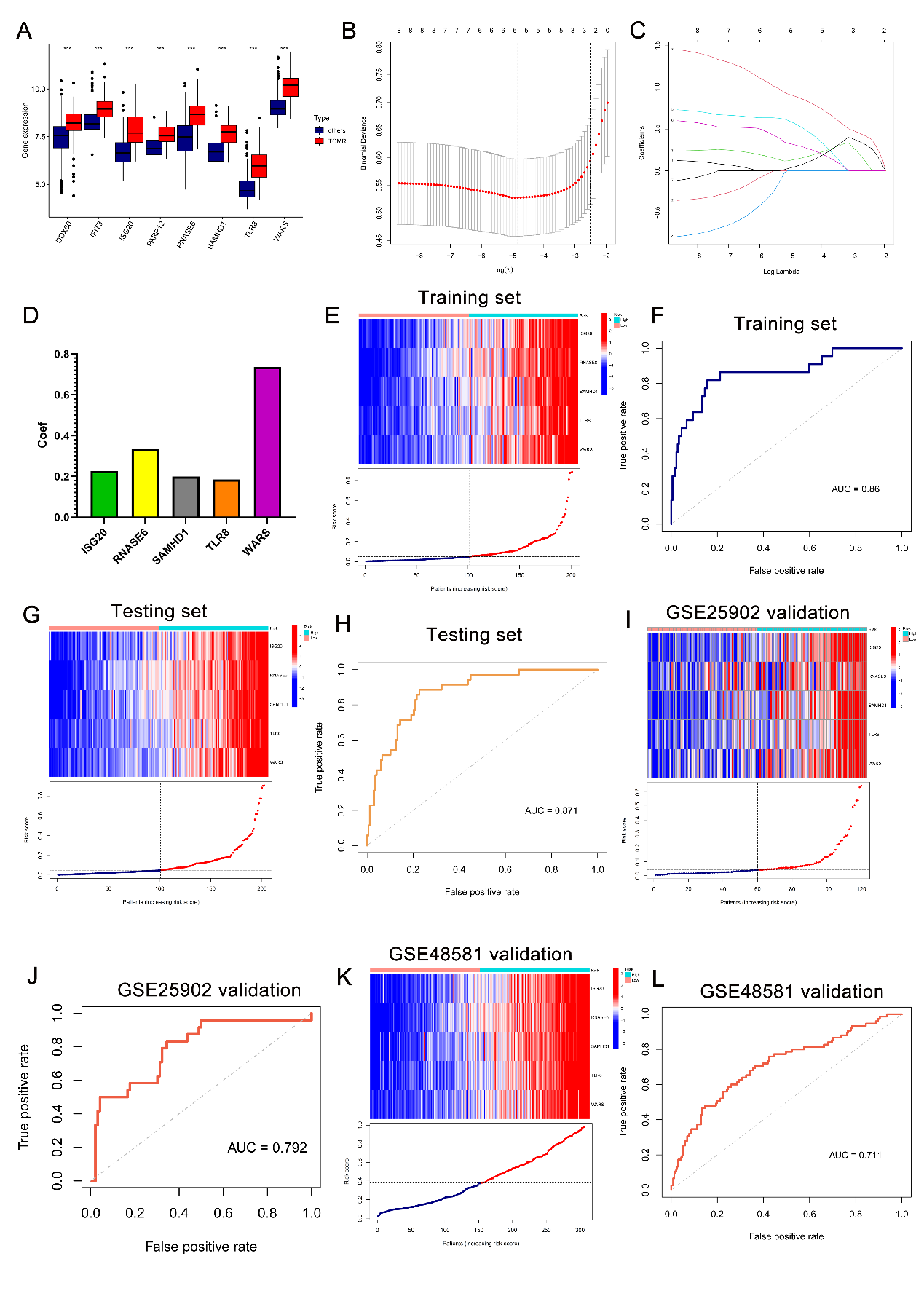


**Figure S2.** Construction and validation of TCMR diagnostic models. (A) Box plots showing expression of DE-RBPs in TCMR (including mixed) and others. (B, C) The 10-fold cross-validation LASSO obtained 5 candidate RBPs. (D) LASSO coefficients of candidate genes. (E-L) Evaluating the performance of the diagnostic model in the training set, the internal testing set and validation sets (GSE25902 and GSE48581). The heatmaps showing gene expression of five genes (ISG20, RNASE6, SAMHD1, TLR8, and WARS) in the model. Patients’ distribution based on the median risk score. ROC curves showing the validity of the model for diagnosing TCMR. ROC, receiver operating characteristic.
